# Optimal enzyme profiles in unbranched metabolic pathways

**DOI:** 10.1098/rsfs.2023.0029

**Published:** 2024-02-09

**Authors:** Elad Noor, Wolfram Liebermeister

**Affiliations:** ^1^ Department of Plant and Environmental Sciences, Weizmann Institute of Science, 76100 Rehovot, Israel; ^2^ Université Paris-Saclay, INRAE, MaIAGE, 78350 Jouy-en-Josas, France

**Keywords:** metabolic pathway, enzymatic rate law, enzyme demand, optimization, protein allocation, bacterial growth law

## Abstract

How to optimize the allocation of enzymes in metabolic pathways has been a topic of study for many decades. Although the general problem is complex and nonlinear, we have previously shown that it can be solved by convex optimization. In this paper, we focus on unbranched metabolic pathways with simplified enzymatic rate laws and derive analytic solutions to the optimization problem. We revisit existing solutions based on the limit of mass-action rate laws and present new solutions for other rate laws. Furthermore, we revisit a known relationship between flux control coefficients and enzyme abundances in optimal metabolic states. We generalize this relationship to models with density constraints on enzymes and metabolites, and present a new local relationship between optimal reaction elasticities and enzyme amounts. Finally, we apply our theory to derive simple kinetics-based formulae for protein allocation during bacterial growth.

## 1. Introduction

The idea that living beings show optimal shapes or behaviour has a very long history. A process like evolution, which combines random mutations with a selection for favourable properties, could potentially lead to optimization, but the question of *if* and/or *when* should we expect living beings to function optimally has been widely debated and is far from solved. In practice, it can be useful to invoke optimality principles to seek insights and design principles that might be relevant in naturally evolved systems [1]. Specifically, cell metabolism has often been studied using this approach [2,3], thanks to the powerful mathematical models that we have to describe it. But although natural selection has been the main inspiration for this study, the evolutionary aspects of pathway optimization are not discussed here, and are rather left for the reader to reflect upon.

Within cells, protein is arguably the most important and central resource, both in terms of contributing to fitness, but also since protein synthesis requires large amounts of energy, metabolic precursors, and ribosomes, and the proteins themselves occupy a significant portion of cellular space. Therefore, a cell should generally save protein wherever it can. This notion, specifically for enzymes, has been mathematically applied in genome-scale metabolic models [4,5], models of core metabolism [6], and in direct comparisons between pathways [7,8]. Since the proteome is a limiting resource, its allocation to different sectors (metabolism, translation, etc.) is a topic of high interest. In some works, people assumed optimality. Other papers assumed a general rule based on linear growth rate control. Interestingly, even a very simple linear chain model, with two reactions representing metabolism and protein synthesis and with a bound on the total protein budget, has been successful in explaining bacterial growth laws and overflow metabolism [9,10]. However, these bacterial growth law models did not consider enzyme kinetics.

Here, we focus on a special case of this cost/benefit analysis: the efficient use of metabolic enzymes in unbranched pathways operating at steady state, giving priority to scenarios that can be solved analytically. We explore several types of kinetic rate laws and introduce the idea of bounding the total metabolite concentration (which is required in some cases for meaningful results). To define states of maximal enzyme efficiency, we can consider two equivalent optimality problems: maximizing a production flux at a given enzyme budget or minimizing protein usage at a given required production flux. In both cases, we maximize the production flux per enzyme usage within the given constraints. If the product of the pathway is directly tied to biomass, the overall enzyme efficiency, called ‘biomass/enzyme efficiency’, can serve as a proxy for cell growth [6]. Furthermore, this optimality problem is also relevant in other contexts, such as metabolic engineering of synthetic pathways using a set of existing and/or new-to-nature enzymes with known kinetic parameters [11,12].

Another perspective often used to analyse metabolic systems is through their control, e.g. the effect of changes in a level of an enzyme on pathway flux [13]. In optimal metabolic states with a bound on the sum of enzyme levels, each enzyme effectively carries an opportunity cost. This cost must be balanced by a marginal benefit, given by the flux control coefficient—as defined in metabolic control analysis (MCA) [13–15]. Hence, for systems in optimal states, there are simple relationships between enzyme abundance and flux control [16–18]. We will recapitulate these results below, generalize them to models with a density constraint on enzyme and metabolite levels, and illustrate them using some of our analytic solutions. In addition, we present a simple rule that links optimal enzyme investments around a given metabolite to the reaction elasticities of this metabolite.

Finally, we show how the analytic solutions derived here might be useful for modelling phenomena on the level of entire cells such as the Monod curve (i.e. the relationship between the concentration of a limiting nutrient and the growth rate of bacteria [19]). We use this model to demonstrate how each kinetic parameter should affect the growth rate under different conditions.

In summary, this paper revisits the question of optimal enzyme allocation and adds to previous results. We focus on unbranched metabolic pathways, extend the optimality problem, discuss new optimality conditions, and present analytic solutions that directly show how different factors determine optimal enzyme levels and fluxes. We discuss general principles, in particular how optimal enzyme levels reflect flux control and local reaction elasticities, and use our theory to derive formulae for kinetics-based bacterial growth.

## 2. Results

### 2.1. Optimal states of unbranched pathways

**Figure 1.**
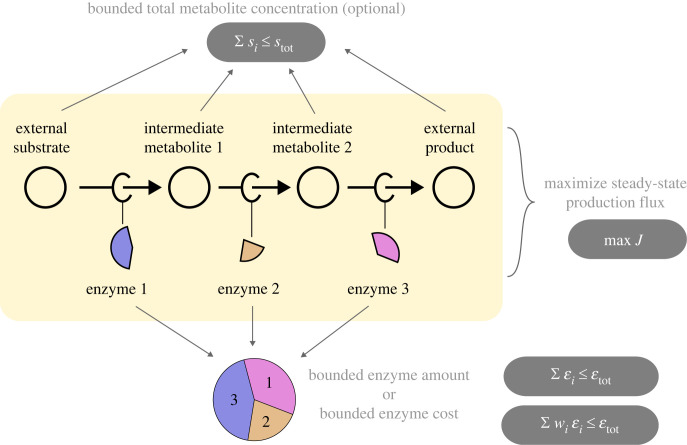
Optimal enzyme levels in unbranched metabolic pathways. In the basic optimality problem in this paper, we consider a chain of reactions and ask how a given protein budget should be spent on metabolic enzymes to achieve a maximal steady-state flux. If the efficiencies (i.e. the reaction rates per catalysing enzyme) of all the enzymes were known, the steady-state flux would determine the enzyme levels, and there would be nothing to optimize. Here we assume that the enzyme efficiencies can be adjusted by choosing the metabolite concentrations (not shown), and we search for the optimal metabolite and enzyme profile. The aim is to maximize the flux at a given total enzyme amount (bottom) and possibly under a constraint on metabolite concentrations (top). The flux ratios between reactions are predefined, for example, assuming equal fluxes in all the reactions (right).

One of the first attempts at analytically solving the enzyme allocation problem was published by Waley [20], who studied short pathways of two to three reactions while assuming that the concentrations of metabolites (which were denoted *linking intermediates*) are much below the enzymes’ *K*_M_ values, and therefore affect the flux linearly. Given the total amount of catalytically active protein (bounded by $\varepsilon_{\mathrm{tot}}$), the relative enzyme concentrations should be such that they maximize rate (figure 1). Based on these assumptions, one can derive simple formulae for the optimal enzyme levels and maximal pathway flux. Later studies repeated this result and generalized it to linear pathways of any size [17,18,21]. Here, we will revisit this general solution and extend it to other types of rate laws beyond the one considered by Waley [20] (which, from now on, we will refer to as *mass-action*).

Consider the following unbranched pathway [22] (figure 1):

2.1 $${\text{S}}_{0}\overset{v_{1}}{⇌}{\text{S}}_{1}\overset{v_{2}}{⇌}\cdots \overset{v_{n}}{⇌}{\text{S}}_{n}.\;$$

In a kinetic model, each variable *s*_*i*_ represents the concentration of a metabolite *i* and each variable $\varepsilon_{i}$ represents the level (molar concentration or mass concentration) of the enzyme catalysing reaction *i*. Imagine that the total enzyme level in the pathway is bounded by $\varepsilon_{\mathrm{tot}}$, i.e.

2.2 $$\sum_{i}\varepsilon_{i}\leq \varepsilon_{\mathrm{tot}}.$$

What would be the optimal strategy for distributing this resource between the reactions in order to maximize the steady-state flux? To answer this question, we need to know how the rate of each reaction depends on the levels of enzyme, substrate, and product, as well as on kinetic parameters. This is described by rate laws. For some rate laws, we can solve the optimization problem and obtain an analytic solution that describes exactly how much of each enzyme should be allocated. Below, we will also consider a variant of this problem with an extra bound on the sum of metabolite levels or with fixed initial and final metabolite concentrations (*s*_0_ and *s*_*n*_).

**Figure 2.**
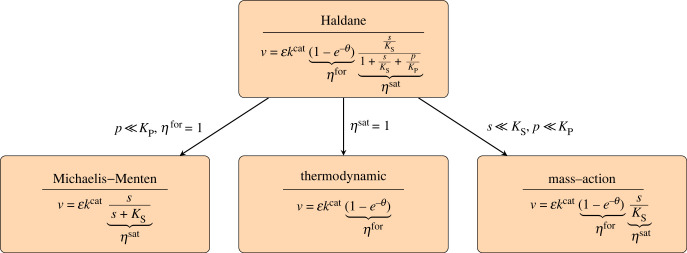
The Haldane rate law and some simplified rate laws. Simplified rate laws are obtained as limiting cases by setting efficiency terms *η*^for^ or *η*^sat^ to 1 or another constant value (figure 3) or by assuming that reactant concentrations are small (in the case of the mass-action rate law). For the enzyme optimality problem in unbranched pathways, we do not know of any analytic solutions for the Haldane rate law. We report here solutions for the other, simplified rate laws (where the solution for the thermodynamic rate law contains an unknown auxiliary parameter). Symbols are explained in the text.

Since single analytic solutions are rare but instructive, we explore them in this article. We consider four different rate laws (summarized in figure 2): the general Haldane rate law (saturable and reversible) which has no analytic solution, and three solvable approximations derived from it. As explained above, the *enzyme levels* $\varepsilon_{i}$ may refer to either molar concentrations or mass concentrations (depending on the modeller’s preference). In the case of molar concentrations, the *k*^cat^ values are catalytic constants (e.g. in units of s^−1^). In the case of mass concentrations, the *k*^cat^ values are specific activities (e.g. in units of μmol × min^−1^ × mg^−1^).

**Figure 3.**
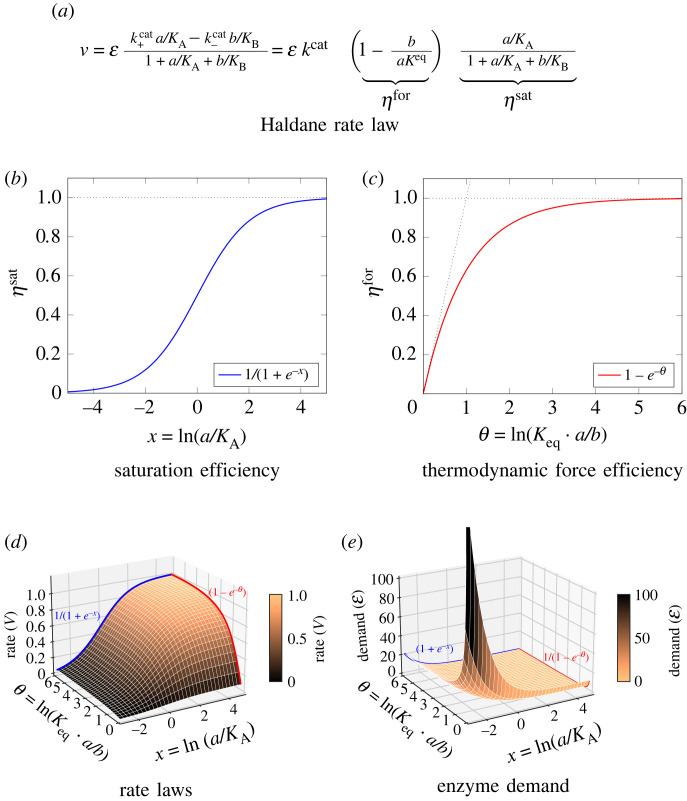
The Haldane rate law and its factorization into efficiency terms. (*a*) The Haldane rate law. In the factorized form (right), thermodynamic and saturation effects are described by separate efficiency factors. (*b*) The saturation efficiency as a function of the substrate concentration (in log-scale, relative to the *K*_A_), assuming *b* ≪ *K*_B_. (*c*) The thermodynamic force efficiency as a function of the driving force *θ*. (*d*) A surface plot showing the rate *v* as a function of *θ* and the substrate concentration (in log-scale, relative to the *K*_A_). The parameters are $K^{\mathrm{eq}}=K_{A}=k^{\mathrm{cat}}=\varepsilon =1$ and *K*_B_ = 10. (*e*) A surface plot showing the enzyme demand for a given rate (*v* = 1). All kinetic parameters are the same as in (*d*).

— *Reversible saturable rate law (Haldane).* As a general rate law for a reaction S ↔ P, we consider the reversible saturable rate law
  2.3 $$v=\varepsilon \frac{k_{+}^{\mathrm{cat}}\;s/K_{S}-k_{-}^{\mathrm{cat}}\;p/K_{P}}{1+s/K_{S}+p/K_{P}},$$
  with Michaelis–Menten constants *K*_S_ and *K*_P_  , which can be factorized into
  2.4 $$v=\varepsilon \;k_{+}^{\mathrm{cat}}\;\underset{\eta^{\mathrm{for}}(\theta (s,p))}{\underbrace{\left(1-e^{-\theta }\right)}}\;\underset{\eta^{\mathrm{sat}}(s,p)}{\underbrace{\frac{s/K_{S}}{1+s/K_{S}+p/K_{P}}}},$$
  with the thermodynamic driving force *θ*(*s*, *p*) = ln(*K*^eq^ *s*/*p*), the thermodynamic force efficiency *η*^for^ and the saturation efficiency *η*^sat^ (figure 3). Note that the driving force can also be defined as −Δ*G*′ which is equal to *RT* ln(*K*^eq^ *s*/*p*), but here we drop the gas constant *R* and temperature *T* to have a unitless (positive) variable. The two efficiency terms can only assume values between 0 and 1. This factorized formulation of the Haldane rate law is equivalent to the one in equation (2.3), based on the constraint that $K^{\mathrm{eq}}=k_{+}^{\mathrm{cat}}/k_{-}^{\mathrm{cat}}\cdot K_{P}/K_{S}$, which is commonly known as the *Haldane relationship* (see [23] for more details). This rate law is the most realistic one that we discuss in this work, and deriving it requires only a few assumptions. However, it is also the most mathematically complex and therefore most questions we raise below do not have analytic solutions. So, in addition, we consider three simplified rate laws as limiting cases.
— *Mass-action rate law.* A very common approximation for enzymatic rate laws (the one also made by Waley [20]) is based on the limit of low metabolite concentrations (*s* ≪ *K*_S_ and *p* ≪ *K*_P_). In this case, the concentration-dependent terms in the denominator (i.e. *s*/*K*_S_ + *p*/*K*_P_) can be neglected and we get
  2.5 $$v=\varepsilon \;(k_{+}^{\mathrm{cat}}\;s/K_{S}-k_{-}^{\mathrm{cat}}\;p/K_{P}).$$
  There are many equivalent ways to write down this rate law. For instance, we can apply the same approximation to the factorized form in equation (2.4) to get $v=\varepsilon \;k_{+}^{\mathrm{cat}}\;(1-e^{-\theta })\;s/K_{S}$, and we can further replace *θ* with its explicit definition based on reactant concentrations and write $v=\varepsilon \;k_{+}^{\mathrm{cat}}/K_{S}\;(s-p/K^{\mathrm{eq}})$. Another common form for this rate law is based on the first-order rate constants $k_{+}\equiv k_{+}^{\mathrm{cat}}/K_{S}$ and $k_{-}\equiv k_{-}^{\mathrm{cat}}/K_{P}$. Using them in equation (2.5) looks like this: $v=\varepsilon \;(k_{+}s-k_{-}p)$. As the rate law resembles mass-action kinetics for non-enzymatic reactions, we will refer to this as the ‘mass-action’ rate law although here the enzyme level appears as a prefactor. Throughout this paper, we will switch between these four different notations based on convenience.
— *Thermodynamic rate law.* If the saturation efficiency *η*^sat^ is approximated by 1 (e.g. in the limit *s* ≫ *K*_S_ and *p* ≪ *K*_P_), we obtain
  2.6 $$v=\varepsilon \;k_{+}^{\mathrm{cat}}\;\left(1-\frac{\;p}{s}\;\frac{1}{K^{\mathrm{eq}}}\right)=\varepsilon \;k_{+}^{\mathrm{cat}}(1-e^{-\theta }).$$
  We will also consider a special case of this rate law where *θ* → 0 and therefore *p*/*s* ≈ *K*^eq^, i.e. the reaction is close to equilibrium. In this case, the rate law becomes $v=\varepsilon \;k_{+}^{\mathrm{cat}}\;\theta$.
— *Irreversible saturable rate law (Michaelis–Menten kinetics).* We next assume that both *p* ≪ *s* *K*^eq^ (which means that *θ* → ∞ and therefore the thermodynamic force efficiency *η*^for^ can be approximated by 1) and also *p* ≪ *K*_P_ (so we can drop *p*/*K*_P_ from the denominator in *η*^sat^). In this case, we obtain the Michaelis–Menten rate law
  2.7 $$v=\varepsilon \;k_{+}^{\mathrm{cat}}\;\frac{s}{s+K_{S}},$$
  which depends on the substrate, but not on the product concentration. Originally, Michaelis & Menten [24] developed this irreversible rate law by assuming that the rate of enzyme–substrate binding is very fast compared to catalysis, and that the catalytic step is irreversible. The assumptions made here lead to the same result but are less stringent.

In the approximations, instead of setting the thermodynamic or saturation efficiencies to their maximal value of 1, we may also approximate them by constant numbers smaller than 1; we obtain exactly the same rate laws, but instead of the *k*^cat^ value we obtain a smaller apparent value in the approximated rate law.

### 2.2. Analytic formulae for unbranched metabolic pathways

How can we characterize metabolic states by using analytic formulae? Here we consider chains of uni–uni reactions, or reactions in which all reactant concentrations except for one substrate and one product are given as model parameters. For such an unbranched pathway with a given type of rate law (e.g. mass-action or Michaelis–Menten rate laws), we are interested in formulae for a number of quantities:

1. *Metabolic steady state.* Given the enzyme levels, external metabolite concentrations and kinetic constants, we can directly compute the stationary fluxes and internal metabolite concentrations. For general metabolic networks, no explicit formulae are known, but for unbranched pathways with some simplified rate laws, explicit formulae exist. Incidentally, this also shows that in these models the steady-state concentrations are unique.
2. *Stability of steady state*. If the Jacobian matrix in a steady state has positive eigenvalues, the state is asymptotically unstable and is not able to persist under the inevitable chemical noise in the cell. Stability is also a prerequisite for metabolic control coefficients being defined. A sufficient (but not necessary) condition for stable steady states in unbranched metabolic pathways is given in electronic supplementary material, section 5.8.
3. *Metabolic control.* The metabolic response coefficients *R*^*X*^ are defined by MCA as the derivatives between steady-state concentrations or fluxes and model parameters (e.g. the enzyme levels). If two model parameters act (exclusively) on the same reaction, all their response coefficients will be the same except for a proportional scaling. Taking this into account, one can define the metabolic control coefficients *C*^*X*^, which describe the same type of derivatives, but for a set of hypothetical, reaction-specific parameters. In practice, if reaction rates are proportional to enzyme levels and if each reaction is catalysed by a single specific enzyme, we can write the enzyme response coefficients as $R_{\varepsilon_{l}}^{X}=C_{l}^{X}\cdot v_{l}/\varepsilon_{l}$. Importantly, throughout this paper we use elasticities, response coefficients, and control coefficients in their unscaled form, for instance unscaled enzyme response coefficients $R_{\varepsilon_{i}}^{J}=\partial J/\partial \varepsilon_{i}$ instead of the common scaled form $(\varepsilon_{i}/J)(\partial J/\partial \varepsilon_{i})=\partial \ln J/\partial \ln\varepsilon_{i}$.
  The metabolic control coefficients can be computed from the stoichiometric matrix and the elasticity matrix, and they satisfy summation and connectivity theorems. They can be computed in two ways: if an analytic formula for the metabolic steady state is known (as is the case in some unbranched pathway models studied below), we may differentiate symbolically by the enzyme levels; otherwise, we may compute the elasticity coefficient matrices by differentiating the rate laws and then compute the control coefficient matrices from them using a known formula; however, since this formula involves a matrix inversion, writing this down as an analytic formulae may be extremely complicated, and control coefficients are usually computed numerically. Symbolic expressions for metabolic control coefficients can be conveniently handled by the PySCeSToolbox [25]. Another way to compute control coefficients, which follows from the enzyme-control rule and works only in optimal states, is described below.
4. *Optimal metabolic states.* In our basic metabolic optimality problem, we define optimal states as states in which a given enzyme budget (a fixed sum of enzyme levels) is allocated to maximize a production flux. Kinetic constants and external metabolites are given, and we compute the optimal metabolite profile, the optimal enzyme profile and the optimal flux. If the flux distribution is known (e.g. a steady-state flux in an unbranched pathway) and increases or decreases proportionally with the enzyme levels, this problem is equivalent to the problem of minimizing the enzyme demand at a given (unit) flux. This convex problem can be solved numerically [26], but analytic solutions were known only for very few cases. Below we present some new analytic solutions. Formulae for optimal enzyme levels and the optimal achievable flux are shown in table 1. We also consider a related problem, maximizing the flux under a constraint on the total enzyme plus metabolite amount.
5. *Metabolic control in optimal states.* The control coefficients tell us how a metabolic system responds to perturbations (resulting in a new state that is stationary, but probably non-optimal). For optimal states (with a constraint on the sum of enzyme levels), the enzyme-control rule states that enzyme levels and flux control coefficients must be proportional. Since we know (from the summation theorem) that the control coefficients must sum to 1, we can conclude: whenever there is an analytic formula for optimal enzyme levels, we also obtain a formula for the control coefficients. Below we extend this to models with general density constraints on enzymes and metabolites, and derive an additional rule that relates enzyme levels to metabolite elasticities around each metabolite.

**Table 1.** A summary of all the rate laws considered in this paper, along with their solutions for optimal enzyme allocation and the resulting minimum pathway cost per flux. The rate laws are roughly ordered by increasing level of complexity. The ‘trivial’ rate law does not have its own section in the text, but is shown here as the limit of the Michaelis–Menten rate law when *s*_*i*−1_ ≫ *K*_M;*i*_, as well as the thermodynamic rate law when $K_{\mathrm{tot}}^{\mathrm{eq}}\;s_{0}/s_{n}\to \infty$. The ‘mass-action’ rate law shown in this table is equivalent to the form used in previous studies [16,17,20]. Here it is shown in a modified form for the purpose of using the same set of kinetic parameters as the other rate laws. In order to calculate the absolute optimal enzyme levels from the relative ones (given in the column titled ‘optimal enzyme allocation’), one can use $\varepsilon_{i}^{*}=\varepsilon_{\mathrm{tot}}(x_{i}/\sum_{\;j}x_{\;j})$ where *x*_*i*_ are the relative values.

name (assumptions)	enzymatic rate law : $v_{i}/\varepsilon_{i}=$	comments/definitions	optimal enzyme allocation: ${\varepsilon_{i}}^{*}\propto$	minimum pathway cost per flux: $\varepsilon_{\mathrm{tot}}/J^{*}=$
trivial (irreversible, saturated)	$k_{i}^{\mathrm{cat}}$	$\alpha_{i}\equiv 1/k_{i}^{\mathrm{cat}}$	*α*_*i*_	||***α***||_1_
Michaelis–Menten (irreversible, saturable)	$k_{i}^{\mathrm{cat}}\cdot \frac{s_{i-1}}{s_{i-1}+K_{M;i}}$	$\beta_{i}\equiv K_{M;i}/k_{i}^{\mathrm{cat}}$ $\sum_{i}s_{i}\leq s_{\mathrm{tot}}$	$\alpha_{i}+\sqrt{\beta_{i}\;\|\beta {\|}_{1/2}}/s_{\mathrm{tot}}$	$\|\alpha {\|}_{1}+\|\beta {\|}_{\frac{1}{2}}/s_{\mathrm{tot}}$
thermodynamic (reversible, unsaturable)	$k_{i}^{\mathrm{cat}}\cdot (1-\frac{s_{i}}{s_{i-1}K_{i}^{\mathrm{eq}}})$	find Ψ s.t. $\prod_{i}\left(\sqrt{\Psi \alpha_{i}}+\sqrt{1+\Psi \alpha_{i}}\right)=\sqrt{\frac{s_{0}}{s_{n}}K_{\mathrm{tot}}^{\mathrm{eq}}}$	$\alpha_{i}\left(\frac{1}{2}+\frac{1}{2}\sqrt{1+{\left(\Psi \alpha_{i}\right)}^{-1}}\right)$	$\sum_{i}\alpha_{i}\left(\frac{1}{2}+\frac{1}{2}\sqrt{1+{\left(\Psi \alpha_{i}\right)}^{-1}}\right)$
thermodynamic (approximate solution)	$k_{i}^{\mathrm{cat}}\cdot (1-\frac{s_{i}}{s_{i-1}K_{i}^{\mathrm{eq}}})$	same as above	no explicit formula	‖***α***‖_1_ · (1 − exp(−(‖***α***‖_1_/‖***α***‖_1/2_)*θ*_tot_))^−1^
mass-action [20] (reversible, unsaturable)	$k_{i}^{\mathrm{cat}}\cdot (1-\frac{s_{i}}{s_{i-1}K_{i}^{\mathrm{eq}}})\cdot \frac{s_{i-1}}{K_{M;i}}$	s_0_ = const, s_n_ = const, $\gamma_{i}\equiv \beta_{i}\cdot \prod_{\;j=i}^{n}K_{\;j}^{\mathrm{eq}}$	$\sqrt{\gamma_{i}}$	$\left\|\gamma {\|}_{\frac{1}{2}}/(s_{0}-\frac{s_{n}}{K_{\mathrm{tot}}^{\mathrm{eq}}}\right)$
Haldane (reversible, saturable)	$k_{i}^{\mathrm{cat}}\cdot (1-\frac{s_{i}}{s_{i-1}K_{i}^{\mathrm{eq}}})\cdot \frac{s_{i-1}/K_{S;i}}{1+\frac{s_{i-1}}{K_{S;i}}+\frac{s_{i}}{K_{P;i}}}$	no known solution, use convex optimization solver		

### 2.3. Optimal metabolic states: analytic solutions

What are the general principles behind optimal enzyme allocation? One important principle, valid in optimal metabolic states, has been shown for pathways with mass-action rate laws [21] and later been confirmed for general rate laws [17]: in optimal states, where the metabolic flux has been maximized at a fixed enzyme budget, enzyme levels and flux control coefficients must be proportional:

2.8 $$\varepsilon_{i}^{*}\propto {C_{i}^{J}}^{*}.$$

Here the star * denotes variables in the optimal state.

To derive this rule, we note that in an optimal state each enzyme has a marginal cost (contribution to the enzyme budget, per mole of enzyme) which must be balanced by the same marginal benefit (contribution to the production flux, per mole of enzyme). If all enzymes contribute to the enzyme budget with equal weights, their marginal costs are the same. This means that also their marginal benefit must be the same, which are given by the (unscaled) flux response coefficients $R_{e_{l}}^{J}=\partial v^{\mathrm{st}}/\partial e_{l}$. This, finally, means that their flux control coefficients $C_{l}^{J}=(\partial v^{\mathrm{st}}/\partial e_{l})/(v^{\mathrm{st}}/e_{l})$ must be proportional to the enzyme levels *e*_*l*_.

Together with the summation theorem for flux control coefficients [14,27], $\sum_{i}C_{i}^{J}=1$, we obtain the conversion formulae

2.9 $$C_{i}^{J}=\frac{\varepsilon_{i}^{*}}{\sum_{\;j}\varepsilon_{i}^{*}}\;\text{and}\;\varepsilon_{i}^{*}=\varepsilon_{\mathrm{tot}}\;C_{i}^{J}.$$

The enzyme-control rule (2.8) provides a condition for metabolic states, independent of the type of rate laws considered. Importantly, it holds only in states of maximal flux, given a fixed total enzyme amount and no other constraints. More realistic models employ also bounds on metabolite levels [5,22,28] for different reasons. First, metabolite levels in real cells are bounded: while metabolite molecules may be small, their concentration is high, and they contribute much more than macromolecules to osmotic pressure. Second, as we will see below, some models without metabolite bounds lead to paradoxical results. We will therefore present a generalized version of the enzyme-control rule that takes metabolite bounds into account.

But what are the general shapes of optimal enzyme profiles, i.e. how do enzyme levels vary across the network? And on what factors (kinetic, thermodynamic or cost factors) does this depend? To answer this, the enzyme-control rule alone would not be enough (because no kinetic details appear in the rule). Also numerical studies would not be enough (because they apply to single cases and yield no general laws). Hence, to study this it would be good to consider analytic solutions. Unfortunately, analytic solutions are not known for general metabolic models, but rather only for special cases of unbranched pathways (such as ones with mass-action rate laws [20]).

We now present analytic formulae for optimal metabolic states with different types of rate laws. We discuss the rate laws in increasing order of difficulty. An overview of all analytic solutions is given in table 1.

#### 2.3.1. Michaelis–Menten rate law

We first consider the Michaelis–Menten rate law (i.e. irreversible reactions with simple saturation kinetics):

2.10 $$v_{i}=\varepsilon_{i}\cdot k_{i}^{\mathrm{cat}}\;\frac{s_{i-1}}{s_{i-1}+K_{M;i}}.$$

In an unbranched pathway at steady state, all rates must be equal. To describe them, we introduce a new variable *J* called the pathway flux, and require that $\forall i\;v_{i}=J$. Now we can use equation (2.10) to find a relationship between substrate and enzyme levels:

2.11 $$\varepsilon_{i}=\frac{J}{k_{i}^{\mathrm{cat}}}\cdot \left(1+\frac{K_{M;i}}{s_{i-1}}\right)=J\left(\alpha_{i}+\frac{\beta_{i}}{s_{i-1}}\right),$$

where we defined $\alpha_{i}\equiv 1/k_{i}^{\mathrm{cat}}$ and $\beta_{i}\equiv K_{M;i}/k_{i}^{\mathrm{cat}}$ for convenience.

Combining this equation with an upper bound on total enzyme from equation (2.2) we get

2.12 $$\varepsilon_{\mathrm{tot}}\geq \sum_{i}\varepsilon_{i}=\sum_{i}J\;\left(\alpha_{i}+\frac{\beta_{i}}{s_{i-1}}\right)=J\left(\sum_{i}\alpha_{i}+\sum_{i}\frac{\beta_{i}}{s_{i-1}}\right),$$

and by rearranging we obtain

2.13 $$J\leq \frac{\varepsilon_{\mathrm{tot}}}{\sum_{i}\alpha_{i}+\sum_{i}\frac{\beta_{i}}{s_{i-1}}}.$$

Maximizing *J* would mean that we reach the upper bound, and therefore we can also treat this as an equality. Since $\varepsilon_{\mathrm{tot}}$ is constant and the only free variables are the metabolite concentrations, the maximal flux is reached when the denominator on the right-hand side is minimized. The problem is that it is a monotonically decreasing function in *s*_*i*_ (for each *i*) and since metabolite concentrations are unbounded, the optimum would be at *s*_*i*_ → ∞, which formally is not a mathematically defined state. But even if we consider this as a limit, the resulting state would not be controllable, but instead very fragile against any changes of enzyme levels (see electronic supplementary material, section 3.1). In reality, of course, the range of physiological osmotic pressures does impose some constraint on the concentrations of small molecules. As a proxy for this effect, we can add another constraint to bound the sum of all metabolite concentrations, $\sum_{i}s_{i}\leq s_{\mathrm{tot}}$. Now, one can show (see electronic supplementary material, section 5.1) that the optimal allocation of enzymes will obey

2.14 $${\varepsilon_{i}}^{*}\propto \alpha_{i}+\sqrt{\beta_{i}}\cdot \frac{\sqrt{\|\beta {\|}_{1/2}}}{s_{\mathrm{tot}}},$$

where ***β*** is the vector of all *β*_*i*_ and $\|x{\|}_{1/2}={\left(\sum_{i}\sqrt{x_{i}}\right)}^{2}$ is the l_1/2_ norm (i.e. $\|\beta {\|}_{1/2}\equiv {\left(\sum_{i}\sqrt{\beta_{i}}\right)}^{2}$). In this case, the maximal flux would be

2.15 $$J^{*}=\varepsilon_{\mathrm{tot}}\cdot {\left(\|\alpha {\|}_{1}+\|\beta {\|}_{1/2}/s_{\mathrm{tot}}\right)}^{-1}.$$

Note that the solution looks essentially the same even if we constrain the first metabolite (*s*_0_) to have a fixed concentration (see electronic supplementary material, section 5.1.4). We will revisit this case in §2.5 in the context of a course-grained model of a growing cell.

If the metabolite density constraint is not very tight (i.e. *s*_tot_ is large enough), we can ignore the second term in equation (2.14), which would be equivalent to assuming that all enzymes are substrate-saturated. In this case, the optimal allocation of enzymes will be proportional to *α*_*i*_ (or inversely proportional to $k_{i}^{\mathrm{cat}}$) and therefore

2.16 $$\lim_{s_{\mathrm{tot}}\to \infty }J^{*}=\varepsilon_{\mathrm{tot}}\cdot {\left(\|\alpha {\|}_{1}\right)}^{-1}.$$

Interestingly, ‖***α***‖_1_, which is equal to the sum of *k*^cat^ reciprocals, is in fact the inverse of the *pathway specific activity* (PSA) as originally defined by Bar-Even *et al.* [11]. Indeed, the idealized scenario considered in that study (where all enzymes were irreversible and saturated) provides an upper bound on the maximal flux achievable. Interestingly, if we see the $\alpha_{i}=1/k_{i}^{\mathrm{cat}}$ as minimal enzymatic turnover times and $\alpha_{\mathrm{PW}}=\varepsilon_{\mathrm{tot}}/J$ is the turnover time of the pathway, we can simply rephrase equation (2.16) by saying that, like in the case of the PSA, turnover times along a pathway are additive. In fact, this holds more generally for any rate law *v*_*i*_ = *e*_*i*_ *k*_*i*_(**s**), if the enzyme turnover times are defined as *τ*_*i*_ = 1/*k*_*i*_, and resembles the fact that in physics, electric resistances in a series of resistors are additive.

One of the reasons the irreversible Michaelis–Menten model is unrealistic is that it does not capture reactions that are close to equilibrium and therefore suffer from a counter-productive reverse flux. This is yet another reason why it might be impossible for some metabolites to reach very high concentrations: they might be products of unfavourable reactions. In most metabolic networks, about half of the reactions are reversible and therefore it would be more realistic to use a reversible rate law such as Haldane’s. However, using the Haldane equation would typically create a system of equations for which no analytic solution is known. So, first, we will consider rate laws that account for the reverse flux but, for simplicity, ignore substrate saturation.

#### 2.3.2. Thermodynamic rate law

Irreversible rate laws like the Michaelis–Menten kinetics depend only on the substrate concentration; in the formula there is no product-dependent reverse term that would decrease the total rate or could make it become negative. However, according to thermodynamics, such laws can only be approximations: thermodynamically feasible rate laws must contain a reverse term and must depend on the thermodynamic imbalance of substrate and product concentrations expressed by the thermodynamic driving force. Some rate laws can even be written as functions of the thermodynamic force alone. Here we describe such a ‘thermodynamic’ rate law, where *v* is proportional to the thermodynamic force efficiency $1-{\text{e}}^{-\theta }$, while the saturation efficiency *η*^sat^ is assumed to be constant and given by 1:

2.17 $$v_{i}=\varepsilon_{i}\;k_{i}^{\mathrm{cat}}(1-e^{-\theta_{i}})=\varepsilon_{i}\;k_{i}^{\mathrm{cat}}\left(1-\frac{s_{i}}{s_{i-1}}\;\frac{1}{K_{i}^{\mathrm{eq}}}\right).$$

This type of kinetics approximates cases where all reactions are saturated (*s*_*i*_ ≫ *K*_M; *i*_) and therefore the thermodynamic force efficiency *η*^sat^ in equation (2.4) becomes 1. The parameters here are the turnover numbers $k_{i}^{\mathrm{cat}}$ and the equilibrium constants $K_{i}^{\mathrm{eq}}$. However, as we will learn soon, the individual equilibrium constants $K_{i}^{\mathrm{eq}}$ do not change the result, and only the overall equilibrium constant ($K_{\mathrm{tot}}^{\mathrm{eq}}=\prod_{i}K_{i}^{\mathrm{eq}}$) matters.

As before, we can define the steady-state pathway flux *J* and apply an upper bound on the total enzyme to get

2.18 $$\begin{array}{cc} & \varepsilon_{\mathrm{tot}}\geq \sum_{i}\varepsilon_{i}=\sum_{i}\frac{J}{k_{i}^{\mathrm{cat}}(1-e^{-\theta_{i}})}=J\cdot \left(\sum_{i}\frac{1}{k_{i}^{\mathrm{cat}}}\cdot \frac{1}{1-e^{-\theta_{i}}}\right)\\ \mathrm{and}\; & J\leq \varepsilon_{\mathrm{tot}}\cdot {\left(\sum_{i}\frac{1}{k_{i}^{\mathrm{cat}}}\cdot \frac{1}{1-e^{-\theta_{i}}}\right)}^{-1}. \end{array}\}$$

Unfortunately, there is no closed-form analytic solution for the maximal rate of this thermodynamic rate law. But we do have something very close which requires rather simple computations. First, we need to invert the functional relationship between the overall driving force (*θ*_tot_) and an auxiliary variable Ψ which is defined by the inverse function of

2.19 $$\ln\left(\frac{s_{0}}{s_{n}}K_{\mathrm{tot}}^{\mathrm{eq}}\right)=\theta_{\mathrm{tot}}=2\sum_{i}\ln\left(\sqrt{\frac{\Psi }{k_{i}^{\mathrm{cat}}}}+\sqrt{1+\frac{\Psi }{k_{i}^{\mathrm{cat}}}}\right).$$

The expression on the right-hand side is analytic and strictly increasing in the range Ψ∈[0,∞), so there is a unique solution that can be found by simple numerical methods. Then, we can use that value to directly calculate the optimal driving forces, enzyme levels, and pathway flux:

2.20 $$\begin{array}{cc} & {\theta_{i}}^{*}=2\ln\left(\frac{1}{k_{i}^{\mathrm{cat}}}\left(1+\sqrt{1+\frac{k_{i}^{\mathrm{cat}}}{\Psi }}\right)\right)+\ln(\Psi k_{i}^{\mathrm{cat}}),\\ & \varepsilon_{i}^{*}=\frac{J^{*}}{2}\cdot \frac{1}{k_{i}^{\mathrm{cat}}}\left(1+\sqrt{1+\frac{k_{i}^{\mathrm{cat}}}{\Psi }}\right)\\ \mathrm{and}\; & J^{*}=2\varepsilon_{\mathrm{tot}}\cdot {\left(\sum_{i}\frac{1}{k_{i}^{\mathrm{cat}}}\left(1+\sqrt{1+\frac{k_{i}^{\mathrm{cat}}}{\Psi }}\right)\right)}^{-1}. \end{array}\}$$

**Figure 4.**
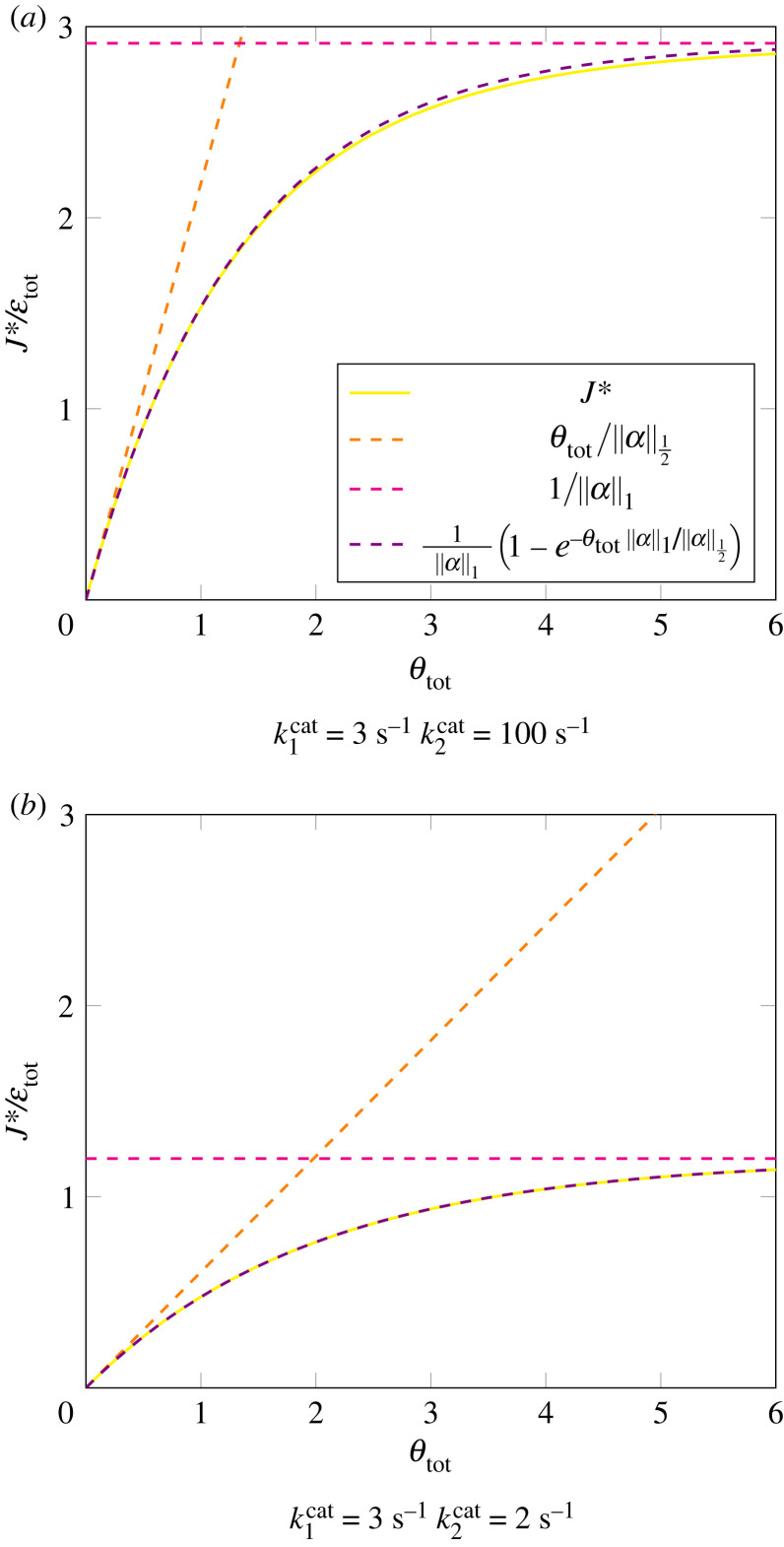
The relationship between the maximal flux per enzyme and the overall driving force for the thermodynamic rate law. Even though we do not have a closed-form solution for J* as a function of *θ*_tot_, we can still plot $J^{*}(\Psi )$ against $\theta_{\mathrm{tot}}(\Psi )$ for varying values of Ψ using equations (2.19) and (2.20). Here, we show this relationship for a pathway with 2 steps (yellow). The parameters are: $K_{i}^{\mathrm{eq}}=1$, $k_{1}^{\mathrm{cat}}=3\;s^{-1}$, and the *k*^cat^ of the second enzyme is either (*a*) $k_{2}^{\mathrm{cat}}=100\;s^{-1}$ or (*b*) $k_{2}^{\mathrm{cat}}=2\;s^{-1}$. The orange dashed line represents the approximations for very small driving forces based on equation (2.23), and similarly in pink for very large driving forces as in equation (2.21). The approximation (dashed purple line) using equation (2.24) is based on effective parameters chosen to match the slope at *θ*_tot_ = 0 and the limiting maximal flux at *θ*_tot_ → ∞.

The full derivation of this solution can be found in electronic supplementary material, section 5.2.2. An example of what the relationship between the driving force and the optimal flux looks like is illustrated in figure 4 for a pathway with two enzymes. Furthermore, a comparison between our exact formula presented here and a numerical solution based on convex optimization [8] shows no difference at all.

These formulae cannot be directly evaluated because of the unknown parameter Ψ. To obtain solutions that do not depend on this parameter, we now consider two limiting cases in which Ψ is either infinitely high (very high driving force) or infinitely low (very low driving force).

When the driving forces are very high (i.e. *θ*_tot_ → ∞), the solution for Ψ in equation (2.19) will approach infinity and the optimal flux becomes

2.21 $$\lim_{\theta_{\mathrm{tot}}\to \infty }J^{*}=\lim_{\Psi \to \infty }\frac{2\;\varepsilon_{\mathrm{tot}}}{\sum_{i}1/k_{i}^{\mathrm{cat}}\cdot \left(1+\sqrt{1+k_{i}^{\mathrm{cat}}/\Psi }\right)}=\frac{\varepsilon_{\mathrm{tot}}}{\sum_{i}1/k_{i}^{\mathrm{cat}}}=\varepsilon_{\mathrm{tot}}\cdot {\left(\|\alpha {\|}_{1}\right)}^{-1},$$

with parameters $\alpha_{i}=1/k_{i}^{\mathrm{cat}}$ defined as before. This solution indeed makes sense as it is equivalent to the fully saturated limit in the Michaelis–Menten case (see §2.3.1, equation (2.16)).

However, when the driving force in the pathway is limited (Ψ has a finite positive value), this driving force will be split between reactions according to their $k_{i}^{\mathrm{cat}}$ values: enzymes with a higher $k_{i}^{\mathrm{cat}}$ value will have to pay a higher penalty due to their driving force being closer to 0. On the other hand, slow enzymes will obtain more driving force, which will help them by having a smaller fraction of reverse flux. Notably, the distribution does *not* depend on the reaction equilibrium constants (only on the overall $K_{\mathrm{tot}}^{\mathrm{eq}}$). Perhaps this is not that surprising if we consider the fact that the concentrations of intermediate substrates and products are unconstrained and therefore we have enough degrees of freedom to adjust to any value of $K_{i}^{\mathrm{eq}}$ as long as the total driving force stays the same.

One can also consider the other extreme where all reactions are close to equilibrium, which means that *θ*_tot_ is close to 0 (and therefore also each *θ*_*i*_ → 0). In this case, we can approximate equation (2.18) by

2.22 $$J\leq \varepsilon_{\mathrm{tot}}\cdot {\left(\sum_{i}\frac{1}{k_{i}^{\mathrm{cat}}}\cdot \frac{1}{1-e^{-\theta_{i}}}\right)}^{-1}\approx \varepsilon_{\mathrm{tot}}\cdot {\left(\sum_{i}\frac{1}{k_{i}^{\mathrm{cat}}\;\theta_{i}}\right)}^{-1}.$$

Maximizing *J* under the constraint $\sum_{i}\theta_{i}=\theta_{\mathrm{tot}}$ yields the following solution (see the full derivation in electronic supplementary material, section 5.2.3):

2.23 $$\begin{array}{cc} & {\theta_{i}}^{*}\approx \theta_{\mathrm{tot}}\;\frac{\sqrt{1/k_{i}^{\mathrm{cat}}}}{\sum_{i}\sqrt{1/k_{i}^{\mathrm{cat}}}}\\ \mathrm{and}\; & J^{*}\approx \frac{\theta_{\mathrm{tot}}\;\varepsilon_{\mathrm{tot}}}{{\left(\sum_{i}\sqrt{1/k^{\mathrm{cat}}}\right)}^{2}}=\varepsilon_{\mathrm{tot}}\cdot \frac{\theta_{\mathrm{tot}}}{\|\alpha {\|}_{1/2}}. \end{array}\}$$

Using the two limiting cases (*θ*_tot_ → ∞ and *θ*_tot_ ≈ 0), we can approximate the solution from equation (2.20) by a much simpler formula, which has the same shape as the thermodynamic rate law for a single reaction:

2.24 $$J^{*}\approx \frac{\varepsilon_{\mathrm{tot}}}{\|\alpha {\|}_{1}}\left(1-\exp\left(-\frac{\|\alpha {\|}_{1}}{\|\alpha {\|}_{1/2}}\theta_{\mathrm{tot}}\right)\right).$$

Figure 4 compares between the precise and approximate solution for two example cases (a more detailed comparison for metabolic pathways of different lengths and parameter choices is shown in electronic supplementary material, section 5.3). One can appreciate that the two curves are nearly indistinguishable, illustrating the good quality of the approximation.

#### 2.3.3. Mass-action rate law

Next, we consider the same unbranched pathway but assuming mass-action rate laws:

2.25 $$v_{i}=\varepsilon_{i}\;k_{i}^{\mathrm{cat}}/K_{M;i}\;\left(s_{i-1}-\frac{s_{i}}{K_{i}^{\mathrm{eq}}}\right)=\varepsilon_{i}\;\beta_{i}^{-1}\;\left(s_{i-1}-\frac{s_{i}}{K_{i}^{\mathrm{eq}}}\right).$$

As before, we defined $\beta_{i}\equiv K_{M;i}/k_{i}^{\mathrm{cat}}$. Note that instead of equation (2.5) we use a form of the mass-action rate law that does not require the turnover rate and *K*_M_ of the product (and instead uses $K_{i}^{\mathrm{eq}}$), to avoid confusing indexation of forward and backward parameters.

Like with the previous rate laws, we define the pathway flux *J* and apply an upper bound on the total enzyme levels:

2.26 $$\begin{array}{cc} & \varepsilon_{\mathrm{tot}}\geq \sum_{i}\varepsilon_{i}=\sum_{i}\frac{J\;\beta_{i}}{s_{i-1}-s_{i}/K_{i}^{\mathrm{eq}}}=J\sum_{i}\frac{\beta_{i}}{s_{i-1}-s_{i}/K_{i}^{\mathrm{eq}}}\\ \mathrm{and}\; & J\leq \varepsilon_{\mathrm{tot}}\cdot {\left(\sum_{i}\frac{\beta_{i}}{s_{i-1}-s_{i}/K_{i}^{\mathrm{eq}}}\right)}^{-1}. \end{array}\}$$

Again, we maximize the flux at a constrained total enzyme level. Optimization with Lagrange multipliers yields the following expressions for the optimal individual enzyme levels (${\varepsilon_{\;j}}^{*}$) and the maximal flux (J*) (see full derivation in electronic supplementary material, section 5.4):

2.27 $$\begin{array}{cc} & \varepsilon_{\;j}^{*}=\varepsilon_{\mathrm{tot}}\;\sqrt{\frac{\gamma_{\;j}}{\|\gamma {\|}_{1/2}}}\\ \;\mathrm{and}\; & J^{*}=\varepsilon_{\mathrm{tot}}\cdot \frac{s_{0}\;K_{\mathrm{tot}}^{\mathrm{eq}}-s_{n}}{\|\gamma {\|}_{1/2}} \end{array}\}$$

where *γ*_*j*_ is defined as

2.28 $$\gamma_{i}\equiv \beta_{i}\prod_{\;j=i}^{n}K_{\;j}^{\mathrm{eq}}.$$

Of course, the exact value of this maximum depends on all the different system parameters. However, it is interesting to consider a naive assumption where all the *γ*_*j*_ parameters are identical. In such a case, the flux in the pathway would decrease quadratically with the number of steps [13]. Of course, we know that in real metabolic pathways the equilibrium constants are typically not close to 1, and therefore this approximation might not have many applications in biology.

What would happen if a mutation improved the catalytic rate of only one of the enzymes $\varepsilon_{i}$ by a factor of *a* (i.e. *β*_*i*_ decreases by a factor of *a*, but the equilibrium constant $K_{i}^{\mathrm{eq}}$ remains the same)? In this case, *γ*_*i*_ would be divided by *a* but the optimal enzyme concentration for this reaction ${\varepsilon_{i}}^{*}$ would only decrease by a factor of $\sqrt{a}$. This saving would then be distributed proportionally among all the *n* enzymes and would contribute to an increase in the pathway flux *J*. On the flip side, if for some reason the activity of one enzyme is decreased by a multiplicative factor *b*, it would need to ‘pay’ (increase the enzyme’s concentration) only by a factor $\sqrt{b}$, and this increase would be ‘funded’ by all of the enzymes together in order to keep the same $\varepsilon_{\mathrm{tot}}$, thus lowering *J*.

#### 2.3.4. Haldane rate law

So far we analysed three cases where all enzymes could be described by the same kinetic rate law: mass-action, Michaelis–Menten, or thermodynamic. Although these rate laws can reliably describe some enzymes in specific conditions, it is very unlikely that such an approximation would apply to *all* the reactions in a single pathway (except for the trivial case of a 1-reaction pathway). A more realistic model would allow all reactions to be reversible and saturable. Here, we will analyse such a case based on the factorized rate law with one substrate and one product. Note that although it is equivalent to equation (2.3), we prefer this formulation because it is easier to separate thermodynamics from saturation effects.

2.29 $$v_{i}=\varepsilon_{i}\cdot k_{i}^{\mathrm{cat}}\cdot (1-e^{-\theta_{i}})\cdot \frac{s_{i-1}/K_{S,i}}{1+s_{i-1}/K_{S,i}+s_{i}/K_{P,i}}.$$

As always, we can assume that all fluxes are equal to *J*, and use the total enzyme budget to get an upper bound:

2.30 $$\begin{array}{cc} & \varepsilon_{\mathrm{tot}}\geq \sum_{i}\varepsilon_{i}=\sum_{i}\frac{J}{k_{i}^{\mathrm{cat}}\cdot (1-e^{-\theta_{i}})\cdot \frac{s_{i-1}/K_{S,i}}{1+s_{i-1}/K_{S,i}+s_{i}/K_{P,i}}}\\ & \;=J\cdot \left(\sum_{i}\frac{1}{k_{i}^{\mathrm{cat}}}{\left(1-e^{-\theta_{i}}\right)}^{-1}\left(1+\frac{K_{S,i}}{s_{i-1}}+\frac{s_{i}K_{S,i}}{s_{i-1}K_{P,i}}\right)\right)\\ \mathrm{and}\; & J\leq \varepsilon_{\mathrm{tot}}\cdot {\left(\sum_{i}\frac{1}{k_{i}^{\mathrm{cat}}}{\left(1-e^{-\theta_{i}}\right)}^{-1}\left(1+\frac{K_{S,i}}{s_{i-1}}+\frac{s_{i}K_{S,i}}{s_{i-1}K_{P,i}}\right)\right)}^{-1}, \end{array}\}$$

where we can now appreciate how this is a generalization of both equations (2.13) (Michaelis–Menten) and (2.18) (thermodynamic). We can see that the maximal pathway flux would be realized when the term in parentheses is minimized with respect to the *s*_*i*_, i.e.

2.31 $${\text{minimize}}_{s}\;\sum_{i}\frac{1}{k_{i}^{\mathrm{cat}}}{\left(1-e^{-\theta_{i}(s)}\right)}^{-1}\left(1+\frac{K_{S,i}}{s_{i-1}}+\frac{s_{i}K_{S,i}}{s_{i-1}K_{P,i}}\right).$$

Unfortunately, as we already mentioned before, no analytic solution to this problem is known in the general case.

### 2.4. Optimal metabolic states: insights from metabolic control analysis

#### 2.4.1. Enzyme-control rule and enzyme-elasticity rule

We now continue with some observations about the enzyme-control rule. The enzyme-control rule equation (2.8) states that enzyme levels and flux control coefficients in optimal states are proportional: $\varepsilon_{i}^{*}\propto {C_{i}^{J}}^{*}$. When the aim is to compute control coefficients in optimal states (at a given enzyme budget $\varepsilon_{\mathrm{tot}}$), this rule comes in handy. The summation theorem tells us that the flux control coefficients must sum to 1; therefore, the optimal enzyme levels are given by the flux control coefficients, multiplied by the (predefined) total enzyme level, and we obtain the simple conversion $C_{i}^{J*}=\varepsilon_{i}^{*}/\varepsilon_{\mathrm{tot}}$ and $\varepsilon_{i}^{*}=\varepsilon_{\mathrm{tot}}\;C_{i}^{J*}$. In the basic form of the rule, we put a constraint on the enzyme mass and assume that enzyme levels are mass densities. If the enzyme levels are molar concentrations, and differently weighted in the constraint, the weights can be taken into account by modifying the rule.

To what types of optimality problems does the rule apply? If the metabolic system is not an unbranched pathway but a general network with one target flux, the rule will still hold (where the flux control coefficients refer to this target flux). This also holds for models with metabolite dilution. However, there are some limitations. Obviously, the rule cannot hold in states in which control coefficients are not defined. This condition may seem unproblematic, but it is actually violated in the pathway with Michaelis–Menten rate laws, because in the limit *s*_*i*_ → ∞ required by optimization, any variations of single enzyme levels would break the steady state. We discuss this in electronic supplementary material, section 3.1.

One way to avoid this problem is to add a bound on metabolite levels. For example, we may consider a generalized density constraint on enzyme levels and metabolite concentrations

Maximize z⋅vs.t. a⋅ε+b⋅s≤ρ,

with a linear flux objective z⋅v instead of a single target flux, and enzyme weights *a*_*l*_, metabolite weights *b*_*i*_, and an upper bound *ρ* on the molecule density. Problems with this constraint lead to a generalized form of the enzyme-control rule. For an unbranched pathway with equal weights *a* for enzymes and equal weights *b* for metabolites (e.g. molecular masses), we obtain the new enzyme-control rule

2.32 $$\varepsilon_{l}^{*}=\varepsilon_{\mathrm{tot}}^{*}\;C_{l}^{J*}-J^{*}\;\frac{b}{a}\;\sum_{i}C_{\;l}^{\;s_{i}*},$$

where the symbols $C_{\;l}^{\;J*}$ and $C_{\;l}^{\;s_{i}*}$ denote, respectively, the flux and control coefficients in the optimal state. Likewise, $\varepsilon_{\mathrm{tot}}^{*}$ is the (non-fixed) sum of enzyme levels emerging in the optimal state. For derivation and details, see electronic supplementary material, section 3.2.

The original enzyme-control rule has a direct and useful consequence. Since enzyme levels (in optimal states) are proportional to flux control coefficients, they need to satisfy a connectivity theorem. Connectivity theorems relate the elasticities of a given metabolite *i* to control coefficients of the reactions around this metabolite. In the case of flux control coefficients, the right-hand side of the theorem is zero, and we obtain a simple equation for the optimal enzyme levels around a metabolite *i*:

2.33 $$\sum_{\;j}\varepsilon_{\;j}\cdot E_{\;j,i}=0,$$

where *E*_*j*,*i*_ = ∂*v*_*j*_/∂*s*_*i*_ denotes the unscaled elasticity between metabolite *i* and reaction *j*.

We call this formula the enzyme-elasticity rule (see electronic supplementary material, section 4). A similar rule for small adaptations of enzyme levels, instead of the enzyme levels themselves, had previously been shown in [29]. For a linear pathway, the enzyme-elasticity rule yields a simple result: in an optimal state, for each metabolite *i* and its producing reaction *j* = *i* − 1 and its consuming reaction *j = i*, the ratio of enzyme levels $\varepsilon_{i}/\varepsilon_{\;j}$ must be equal to the absolute inverse ratio of the elasticities $\left|E_{c_{i}}^{v_{\;j}}/E_{c_{i}}^{v_{i}}\right|$. An example of this, with the mass-action rate law, is derived in electronic supplementary material, section 4. In contrast to the enzyme-control rule (which yields only one equation for the entire pathway), the enzyme-elasticity rule yields an equation for every internal metabolite, which determines the ratio of the enzyme levels around this metabolite. Together with the known sum of enzyme levels, these rules determine the enzyme profile completely (given all the elasticities in the optimal state).

#### 2.4.2. Analytic formulae for metabolic control

We learned that enzyme levels in optimal states (with a bound on total enzyme, and maximizing the steady-state flux) satisfy two general laws, the enzyme-control rule and the enzyme-elasticity rule. Hence, if we have a formula for the optimal enzyme levels, and if the enzyme-control rule applies, we can compute the flux control coefficients. Moreover, the enzyme-elasticity rule (which comes from the connectivity theorem) relates the optimal enzyme levels to elasticities. These formulae can be trusted, but for didactic reasons we set out to compute the control coefficients for some of the rate laws analytically (also demonstrating different ways to compute them) and compared them to optimal enzyme levels. Here we summarize this briefly; details can be found in electronic supplementary material, section 5.

— *Michaelis–Menten rate law*. In metabolic pathways with irreversible rate laws, the first reaction has usually full flux control and therefore a flux control coefficient of 1, while the remaining reactions of no flux control and therefore flux control coefficients of 0. But under an optimization, this changes. All metabolite levels go to infinity and all elasticities go zero. Mathematically, the optimal state does not exist (because infinite concentrations are not real numbers), but even if we assume that enzymes could be saturated completely, any enzyme variation would break the steady state. This means that the control coefficients are not defined, and so the enzyme-control rule does not apply (see electronic supplementary material, section 3.1). To obtain meaningful results, we therefore studied an optimality problem with additional metabolite constraints. In this case, metabolite levels remain finite and the control coefficients remain defined.
— *Thermodynamic rate law.* For pathway models with the thermodynamic rate law, we derived a simple formula for the flux control coefficients that contains only the enzyme levels, the *k*^cat^ values, and the flux. Even if we do not have a closed formula for the flux as a function of enzyme levels, we can obtain a closed formula for the flux control coefficients by using a trick. From the rate law, we first obtain a relationship between enzyme levels, external metabolite levels, kinetic constants and flux:
  2.34 $$\prod_{i=1}^{n}\left(1-\frac{J}{\varepsilon_{i}k_{i}^{\mathrm{cat}}}\right)=\frac{s_{n}}{s_{0}}\frac{1}{K_{\mathrm{tot}}^{\mathrm{eq}}}\;$$
  (see equation (S31) in electronic supplementary material, section 5.2.1, for the derivation). While we cannot solve this for the flux *J* directly, we can obtain the response coefficients $R_{\varepsilon_{l}}^{J}=\partial J/\partial \varepsilon_{l}$ by implicit differentiation. This yields the flux control coefficients (derivation in electronic supplementary material, section 5.3.2):
  2.35 $$C_{l}^{J}=\frac{{\left(\varepsilon_{l}\;k_{l}^{\mathrm{cat}}-J\right)}^{-1}}{\sum_{i=1}^{n}{\left(\varepsilon_{i}\;k_{i}^{\mathrm{cat}}-J\right)}^{-1}}.$$
  The control coefficients are proportional to ${\left(\varepsilon_{l}\;k_{l}^{\mathrm{cat}}-J\right)}^{-1}$ and normalized to a sum of 1 as required by the summation theorem. For didactical reasons, we checked the connectivity theorem (electronic supplementary material, section 5.3.1) and the enzyme-control rule (electronic supplementary material, section 5.3.4).
— *Mass-action rate law.* With the reversible mass-action rate law, we can analytically compute the flux control coefficients and verify that they satisfy summation and connectivity theorems. To verify that the enzyme-control rule is satisfied in the optimal state, we use the explicit formula (2.26) for the steady-state flux and take derivatives to obtain the flux control coefficients:
  2.36 $$C_{l}^{J}=\frac{\gamma_{l}/\varepsilon_{l}}{\sum_{\;j}\gamma_{\;j}/\varepsilon_{\;j}},$$
  with *γ*_*l*_ defined as above (derivation in electronic supplementary material, section 5.4.2). The coefficients sum to 1 as required by the summation theorem. Again, we verified the connectivity theorem (electronic supplementary material, section 5.4.4) and the enzyme-control rule (electronic supplementary material, section 5.4.5) for didactical reasons.

### 2.5. A cell model with enzyme kinetics and metabolite constraints

As an illustrative example, we now apply our formulae to a simple model of growing cells, describing transport processes, metabolism and macromolecule synthesis, respectively, by lumped reactions. Obviously, none of the rate laws considered throughout this work can fully capture the dynamics of growing cells. For example, having only one single reaction representing metabolism is a gross oversimplification (and likewise for transport and translation). Nevertheless, we might still be able to draw insights from this model if we make the right assumptions. This approach has been successful in the past: by considering simple cell models and assuming that enzyme efficiencies are constant (i.e. completely independent of growth rate and metabolite concentrations), Basan *et al.* [10] were able to show how overflow metabolism in *E. coli* corresponds to a proteome-efficient allocation of enzymes. Here we ask how the predictions would change by accounting for enzyme kinetics. Instead of assuming that enzyme efficiencies are constant and given, we consider a model in which protein allocation and metabolite concentrations are always optimized in order to maximize the growth rate.

**Figure 5.**
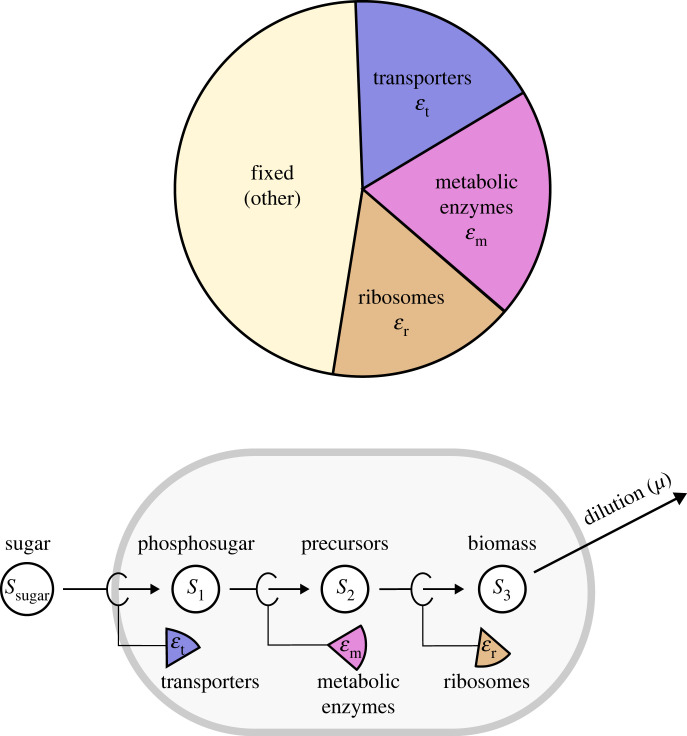
A growing cell described as a chain of reactions. Top: Inspired by the cell model in [9] explaining ‘bacterial growth laws’, we assume a fixed protein budget consisting of a fixed protein fraction and variable fractions for transporters, metabolic enzymes, and ribosomal proteins, but with a fixed sum. The proportions of the variable fractions are chosen to maximize growth. Bottom: The growing cell is described as a chain of reactions, each catalysed by one of the protein fractions. For a constant biomass production, the three reactions must be in steady state.

In our simple cell model (figure 5), production processes are represented by an unbranched chain of reactions, describing overall transport, overall metabolism, and protein synthesis. The steady-state fluxes are proportional to the cell growth rate and optimized under a constraint on the sum of enzyme levels ($\varepsilon_{\mathrm{tot}}$) and another one on metabolite levels (*s*_tot_). For convenience, in this section we will replace the pathway flux J* with the standard symbol *μ* for growth rate. It should be noted that dilution of the intermediate compounds is ignored even though in reality some of them can have a high total concentration (e.g. the precursors in *S*_2_) and thus a considerable growth dilution rate which affects protein allocation strategies.

For our model, we chose to use the Michaelis–Menten approximation, which gives the most realistic results (although we also discuss the two other analytical solutions at the end of this section). Metabolite concentrations (as extra variables) can be adjusted and become part of the optimization problem. We then apply the formulae derived in the previous sections of this paper to find the optimal allocation of enzymes and thereby maximize the growth rate of the cell. Importantly, all calculations are completely based on analytic expressions.

In §2.3.1, we derived a formula for the optimal allocation and maximal flux in the case where all metabolite levels are free variables (including *s*_0_, which is denoted here by *s*_sugar_). However, since in this cell model *s*_sugar_ represents the concentration of an external nutrient that is not subject to optimization, we would like to treat it as a constant system parameter (and later show how the optimum responds to changes in it). Fortunately, adding this assumption changes the optimal solution only slightly, as described in electronic supplementary material, section 5.1.4. The optimal growth rate as a function of *s*_sugar_ can be written in the following simple form:

2.37 $$\mu =\mu^{\max}\cdot \frac{s_{\mathrm{sugar}}}{s_{\mathrm{sugar}}+K_{\mathrm{Monod}}},$$

where we define

2.38 $$\begin{array}{cc} & \mu^{\max}\equiv \frac{\varepsilon_{\mathrm{tot}}}{1/k_{t}^{\mathrm{cat}}+1/k_{m}^{\mathrm{cat}}+1/k_{r}^{\mathrm{cat}}+{\left(\sqrt{K_{M;m}/k_{m}^{\mathrm{cat}}}+\sqrt{K_{M;r}/k_{r}^{\mathrm{cat}}}\right)}^{2}/s_{\mathrm{tot}}}\\ \mathrm{and}\; & K_{\mathrm{Monod}}\equiv \frac{K_{M;t}/k_{t}^{\mathrm{cat}}}{1/k_{t}^{\mathrm{cat}}+1/k_{m}^{\mathrm{cat}}+1/k_{r}^{\mathrm{cat}}+{\left(\sqrt{K_{M;m}/k_{m}^{\mathrm{cat}}}+\sqrt{K_{M;r}/k_{r}^{\mathrm{cat}}}\right)}^{2}/s_{\mathrm{tot}}}. \end{array}\}$$

As implied by the symbol for *K*_Monod_, this form corresponds to empirical observations by Monod [19] (and further followed up by others [30]) who stated that growth rate increases with the nutrient concentration until reaching a saturation level where growth is fastest. Interestingly, the value of *K*_Monod_ (e.g. the level of *s*_sugar_ for which growth rate is half of its maximum) is not determined solely by the kinetic parameters of the transporter.

**Figure 6.**
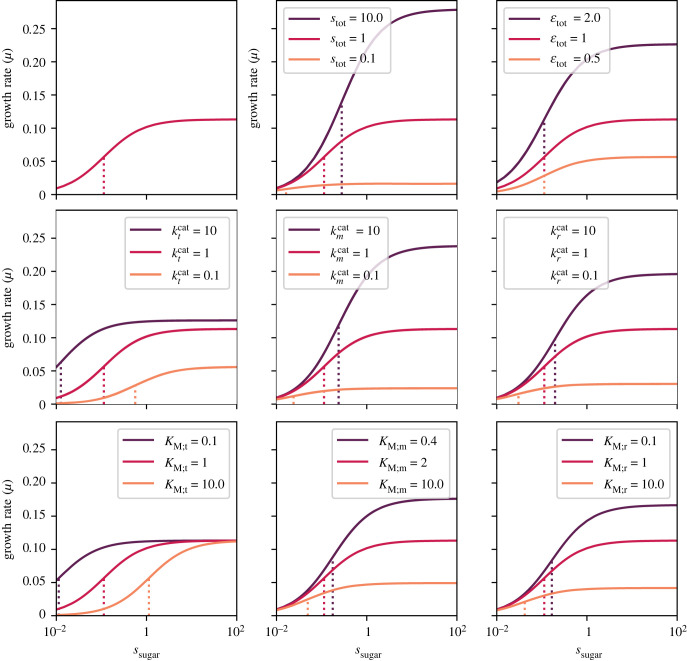
The optimal achievable flux in a cell model—i.e. the growth rate *μ*—for a given total enzyme $\varepsilon_{\mathrm{tot}}$. The model contains three irreversible steps based on Michaelis–Menten kinetics. The default parameters are $k_{t}^{\mathrm{cat}}=k_{m}^{\mathrm{cat}}=k_{r}^{\mathrm{cat}}=K_{M;t}=K_{M;r}=1$, *K*_M;m_ = 2, and the upper bounds are set to *s*_tot_ = 1, $\varepsilon_{\mathrm{tot}}=1$. The panel on the upper-left corner shows the maximal flux as a function of *s*_sugar_ for the default parameters. In each other panel, only one of the parameters is changed. The vertical dotted lines mark the *K*_Monod_ (i.e. the concentration of *s*_sugar_ where the flux is 50% of the maximum). Note that the *x*-axis shows values on log-scale, and therefore the curves have a sigmoidal shape (rather than the familiar Monod-style). See electronic supplementary material, figure S1, for plots of the enzyme demands.

To better understand how changes in the model parameters affect cell growth, we considered a toy example where all constants are set to a default value of 1 (except for *K*_M;m_ = 2). In figure 6, we plot the value of *μ* as a function of *s*_sugar_ (based on equations (2.37)–(2.38)), each time changing one of the parameters. In almost all cases, we find a trade-off between growth and binding affinity, namely that improving the kinetics (or relaxing a constraint) improves the maximal growth rate while also increasing *K*_Monod_ (i.e. making it worse since higher *K*_Monod_ means higher a concentration of sugar is required to reach the same growth rate). The three exceptions are the response to changes in $\varepsilon_{\mathrm{tot}}$ which only affects *μ*^max^, in *K*_t_ which only affects the *K*_Monod_, and in $k_{t}^{\mathrm{cat}}$ which can improve both growth parameters at the same time. Indeed, by observing the formulae in equation (2.38), one can see that *μ*^max^ and *K*_Monod_ are proportional, with a ratio given by $\varepsilon_{\mathrm{tot}}/(K_{M;t}/k_{t}^{\mathrm{cat}})$. Hence, all parameters that are only in the denominator should affect *μ*^max^ and *K*_Monod_ in the same fashion (which leads to a trade-off), while the three parameters in the numerators have each their own unique effect.

**Figure 7.**
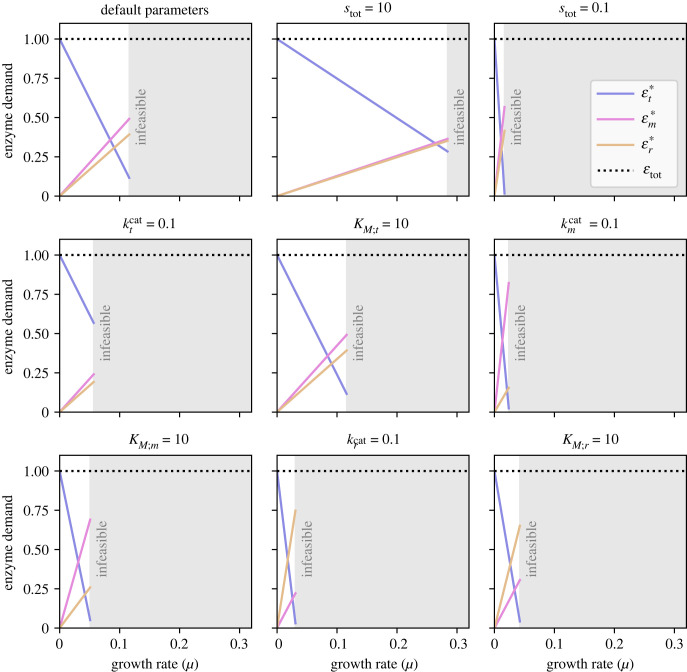
The sizes of the three enzyme sectors ($\varepsilon_{t}^{*}$, $\varepsilon_{m}^{*}$ and $\varepsilon_{r}^{*}$) as a function of the flux (J*) when the allocation is optimal. The default parameters are $k_{t}^{\mathrm{cat}}=k_{m}^{\mathrm{cat}}=k_{r}^{\mathrm{cat}}=1$, *K*_*M;t*_ = *K*_*M;r*_ = 1, *K*_*M;m*_ = 2 (we chose another value in order prevent the curves for $\varepsilon_{m}$ and $\varepsilon_{r}$ from overlapping), and the upper bounds are set to *s*_tot_ = 1, $\varepsilon_{\mathrm{tot}}=1$. In each panel, only one of these parameters is changed. The grey shaded area marks growth rates that are higher than the maximum, *μ*^max^. The linear protein allocation curves are caused by the irreversible transport step. In the optimization, this leads to constant optimal metabolite concentrations, and the protein fractions $\varepsilon_{m}^{*}$ and $\varepsilon_{r}^{*}$ must change proportionally with the flux. The line slopes are given by the enzyme efficiencies at the optimal metabolite concentrations, and the transporter fraction—the residual of the protein budget—must be a falling straight line.

This result might have implications for the design of optimal enzyme regulation. As the rate of sugar transport is not affected by any of the metabolite concentrations inside the cytoplasm, the transport reaction acts as an information buffer in the sense that any changes in the external sugar concentration do not affect the optimal allocation of enzymes (and metabolites) within the cytoplasm. The only thing that changes is the relative abundance of the transporter versus all other (cytoplasmic) enzymes. This rule becomes clear when plotting the optimal enzyme allocations as a function of the pathway flux (i.e. the growth rate), as shown in figure 7. The two cytoplasmic fractions (metabolic enzymes and ribosomes) change proportionally and appear as straight lines that cross the axis origin. Since $\varepsilon_{\mathrm{tot}}$ is a constant, the remainder (i.e. the level of the transporter) also decreases linearly with growth rate. These three straight lines extend only up to a point (where *μ* = *μ*^max^). This limit represents the fully saturated transporter, which has an efficiency of $k_{m}^{\mathrm{cat}}$ and therefore an optimal abundance of $\mu^{\max}/k_{m}^{\mathrm{cat}}$. Higher concentrations of *s*_sugar_ would not generate any further benefits.

In fact, the principle described above can be shown to be true in a much more general case. As long as one section of the metabolic network is ‘isolated’ from the rest (i.e. connected only by irreversible steps), changes in the upstream parameters will affect the incoming flux, but would not change the optimal allocation of enzymes (and also not the metabolite concentrations). Curiously, in such cases our results predict, based on an underlying kinetic model, that the optimal protein efficiencies are independent of the external glucose concentration and, as a consequence, protein fractions vary linearly. Thus, our model predictions are very similar to ones from previous proteome allocation models that are based on empirical observations.

A series of irreversible steps is only one option for a simple model of cell growth. Alternatively, one might consider cells living in an environment that provides high metabolite concentrations, albeit a limited overall thermodynamic driving force. In this case, applying our thermodynamic rate law with the approximation from equation (S46) in the electronic supplementary material, section 5.3, yields another simple growth formula:

2.39 $$\begin{array}{rl} \mu (s_{\mathrm{sugar}}) & =\mu^{\max}\left(1-{\left(\frac{A}{s_{\mathrm{sugar}}}\right)}^{B}\right)\\ \mathrm{and}\;\;\;\mu^{\max} & \equiv \varepsilon_{\mathrm{tot}}{\left(\sum_{i}1/k_{i}^{\mathrm{cat}}\right)}^{-1}, \end{array}\}$$

where $A\equiv s_{n}K_{\mathrm{tot}}^{\mathrm{eq}}$ and $B\equiv (\sum_{i}1/k_{i}^{\mathrm{cat}})/{\left(\sum_{i}1/\sqrt{k_{i}^{\mathrm{cat}}}\right)}^{2}$. Again, the curve parameters depend on all the $k_{i}^{\mathrm{cat}}$ values. In contrast to the Michaelis–Menten based growth formula in equation (2.38), this formula is also sensitive to product concentrations (*s*_*n*_). This may become important close to chemical equilibrium, where product accumulation can decrease growth in a way that cannot be bypassed by any regulatory mechanisms. In this case, our cells would profit from the presence of a ‘cleaner’ strain that removes the accumulating product, which has been proposed as a general mechanism that would encourage symbiosis [31].

Finally, the last option to consider for an analytical cell growth model is based on the mass-action rate law. However, from equation (2.27) we can immediately see that in this case the optimal enzyme fractions are direct functions of the kinetic parameters and do not depend on *s*_0_ or *s*_*n*_. Therefore, when changing the sugar concentration, enzyme allocation will remain constant. Furthermore, the growth rate will be a linear function of *s*_sugar_, without any saturation behaviour. This means there is no way to define *μ*^max^ and *K*_Monod_ for the mass-action case.

## 3. Discussion

In this study, we addressed the enzyme allocation problem in unbranched pathways analytically by considering a number of different rate-law approximations. Along the way, we learned that each approximation comes with its own idiosyncrasies. For instance, a model with Michaelis–Menten kinetics requires an extra metabolite constraint to obtain a valid optimal state, and the ‘thermodynamic’ rate law requires inverting a function in order to find the value of the Lagrange multiplier. Fortunately, there is a very good approximate solution that works well across the entire range of driving forces.

For historical reasons, we solved the optimality problem under the assumption that there is a bound on the sum of enzyme abundances—$\sum_{i}\varepsilon_{i}\leq \varepsilon_{\mathrm{tot}}$—without explicitly specifying the units. The common interpretation would be that $\varepsilon_{i}$ are *molar* concentrations (where the symbol $k_{i}^{\mathrm{cat}}$ represents the turnover numbers in units of 1 over time). However, the total *mass* concentration of all pathway enzymes may be a better proxy for cost, since crowding effects in the cytoplasm are often limiting the total mass concentration of all soluble proteins, including most enzymes. Furthermore, fast-growing cells are often limited by the rate of protein elongation, and the molecular mass is nearly proportional to the gene length. Therefore, we usually think of $\varepsilon_{i}$ as *mass* concentrations (e.g. in g × m^−3^) and of $k_{i}^{\mathrm{cat}}$ as specific activities (e.g. in mol × s^−1^ × g^−1^). Nevertheless, the analytic derivation and solutions provided here are agnostic to the choice of units and are equally valid for both these interpretations. Moreover, one could imagine a completely different set of linear weights for the enzyme cost function (i.e. $\sum_{i}w_{i}\;\varepsilon_{i}\leq \varepsilon_{\mathrm{tot}}$, as in figure 1). If one thinks of the weights as scaling factors for the *k*^cat^ values, the provided solutions will still hold (while making use of the new ‘effective’ *k*^cat^ values).

Previous studies focused on the ‘mass-action’ rate law, justifying it by saying that the general reversible form derived by Haldane can be approximated at the limit of low concentrations. However, it is quite rare to have all the substrates and products of an enzyme at concentrations that are way below their *K*_M_ values [32]. Furthermore, the limit of all reactant concentrations going to zero is not very meaningful because, in the first place, Haldane derived his rate law assuming enzymes are much less abundant than metabolites [33]. Interestingly, just being close to equilibrium is not enough for this approximation, since the product can still affect the rate nonlinearly via *η*^sat^ (figure 2). Here we tried to consider more comprehensively all the different approximations that yield an analytic solution to optimal enzyme allocation in unbranched pathways (table 1).

One of these approximations is the Michaelis–Menten rate law, which is widely used in enzymatic assays and metabolic modelling of irreversible reactions. Curiously, using it for the simple optimality problem with linear chains leads to paradoxical results: the metabolite concentrations go to infinity, the elasticities vanish and flux control coefficients are not defined. For solving this problem, we introduced a new upper bound on the total metabolite concentration in order to obtain realistic results and derive analytic solutions based on this rate law. Although it is very reasonable to assume that concentrations of small molecules (and not just enzymes) are restricted in cells, this fact is often ignored in metabolic models. One of the rare cases where this constraint was taken into account is the work of Dourado *et al.* [34], who found empirical evidence to the fact that there is a balance between enzymes and substrates when minimizing the total mass concentration. In addition to an analytic solution, we also found a new enzyme-control rule for models with a constraint on enzyme and metabolite concentrations: in this case, the enzyme amounts do not only reflect the flux control coefficients, but a sum of flux and concentration control coefficients (see electronic supplementary material, section 3.2).

Besides ‘mass-action’ and Michaelis–Menten kinetics, we discussed a solution for one other rate law which we call *thermodynamic*, as the rate is only affected by metabolite levels through the thermodynamic driving force (i.e. ignoring any saturation effects). One advantage of this rate law is that it does not require knowing the *K*_M_ values (which are often difficult to come by). The thermodynamic-only approximation was also used for justifying the max–min driving force (MDF) method (as described in electronic supplementary material, section 5.3.5), which similarly aims to quantify the efficiency of metabolic pathways [35]. But, unlike the solution presented here, MDF does not explicitly optimize a simplified version of a kinetic rate law, but rather applies a heuristic based on the assumption that the lowest driving forces are mainly responsible for increased enzyme demands, and the total driving force should therefore be as evenly distributed as possible. On the other hand, the advantage of the MDF method is that it takes metabolite concentration bounds into consideration.

All throughout this paper, we only considered pathways with uni–uni reactions (one substrate, one product, with stoichiometric coefficients of 1). In reality, many reactions involve co-factor pairs or other substrates or products. Instead of deriving results for this general case, we assumed that these extra reactants may exist, but with known concentrations. In this case, the rate laws contain extra terms, but these terms can be rearranged to yield the same simple formulae as in the uni–uni case, using *effective* kinetic constants. We demonstrate this for the case of two substrates and two products following convenience kinetics [36] in electronic supplementary material, section 5.6. Notably, this logic also applies to reactions with more than two substrates and products as well as to enzyme activation or inhibition with constant activator or inhibitor levels.

This paper can be seen as an exercise in solving the enzyme allocation problem and describing the optimal states analytically using MCA. Although some might argue that the required approximations are not realistic, they do represent a step forward compared to the very common approach of assuming that metabolites have no effect on enzyme efficiency at all. On the other hand, adding metabolite concentrations as extra variables greatly increases the complexity of models and typically renders them unsolvable. Therefore, the solutions provided here might be handy, as the assumption of metabolite steady state combined with the optimality argument give us analytic expressions that only depend on the initial and final metabolites in the pathway. We demonstrated this result using a toy example for a course-grained model of cell growth, and showed how the analytic solutions provide valuable insights about the effects of changes in each parameter—all this without the need to simulate the metabolic network or use nonlinear solvers. One could imagine a scenario where metabolic engineers, while trying to improve a biochemical pathway, have to decide in which enzyme to invest their time. Although MCA can perfectly serve this purpose while covering more scenarios, it requires tools and language that can seem opaque to people lacking the proper mathematical background. The solutions provided here should be more approachable and serve as simple guidelines while capturing a wider range of cases than previously explored in the literature. We hope that future studies will apply and extend this approach to other, more complex models.

## Data Availability

Accompanying code (Python Jupyter notebooks) is available at https://zenodo.org/doi/10.5281/zenodo.10069509 [37] as well as https://gitlab.com/elad.noor/optimal_enzyme_investment. Supplementary material is available online [38].
